# *Polg* mtDNA mutator mice reveal limited involvement of vertebral bone loss in premature aging-related thoracolumbar hyperkyphosis

**DOI:** 10.1016/j.bonr.2022.101618

**Published:** 2022-08-30

**Authors:** Olivier Roessinger, Thomas Hügle, Ulrich A. Walker, Jeroen Geurts

**Affiliations:** a Department of Rheumatology, Lausanne University Hospital, Avenue Pierre Decker 4, 1005 Lausanne, Switzerland; b Department of Rheumatology, University Hospital of Basel, Petersgraben 4, 4031 Basel, Switzerland

**Keywords:** Aging, Hyperkyphosis, Computed tomography, Vertebral bone

## Abstract

**Background:**

Age-related hyperkyphosis is multifactorial and involves alterations of vertebral bone, intervertebral discs (IVD) and paraspinal muscles. The relative contribution of these tissues remains unclear. Here, we compared differences in vertebral bone microarchitecture and IVD thickness between prematurely aging mice with spinal hyperkyphosis and wild type littermates.

**Methods:**

Thoracolumbar vertebral columns were dissected from homozygous *Polg*^*D257A*^ and age-matched wild type littermates. Micro-computed tomography was performed to quantify cortical and trabecular bone parameters at anterior and posterior portions of T8-L4 vertebrae. In addition, vertebral shape, transaxial facet joint orientation and IVD thickness were quantified. Differences in anterior/posterior ratios between genotypes were compared by Student's *t-*test and association between vertebral bone and IVD parameters was investigated using Pearson correlation analysis.

**Results:**

Hyperkyphotic homozygous mice displayed generalized osteopenia that was more pronounced at the posterior compared with anterior portion of thoracolumbar vertebrae. An increase in the anterior/posterior ratio of trabecular bone parameters was revealed at the thoracolumbar junction (T13-L1). *Polg*^*D257A*^ displayed diffuse loss of cortical bone thickness, yet anterior/posterior ratios were unchanged. Despite generalized and regional bone loss, vertebral shape was unaffected. *PolG*^*D257A*^ mice showed a 10–20 % reduction of IVD thickness at both thoracic and lumbar levels, with only minimal histopathological changes. IVD thickness was negatively correlated with anterior/posterior ratios of trabecular bone parameters, as well as with more coronally oriented facet joints, but negatively correlated with the anterior/posterior ratio of cortical bone thickness.

**Conclusions:**

Aging-induced regional changes of vertebral trabecular and cortical bone did not lead to altered vertebral shape in *Polg*^*D257A*^ mice but may indirectly cause hyperkyphosis through reduction of IVD thickness. These findings suggest a limited role for aging-induced bone loss in spinal hyperkyphosis and warrants further research on the involvement of paraspinal muscle degeneration.

## Highlights

• MtDNA mice exhibit bone loss, mainly in the posterior parts of the vertebral bodies.
• Regional loss of bone in mtDNA mice did not alter vertebral shape.
• Vertebral bone quality may be inversely correlated with intervertebral disc height.
• Vertebral bone loss may be of minor importance in mtDNA mice hyperkyphosis.

## 1 Introduction

Kyphosis is a dorsal convexity of the thoracic and sacral portions of the spine. The gold standard for quantifying thoracic kyphosis is the measurement of the Cobb angle on a standing lateral spine radiograph. In young individuals, normal values of the Cobb angle are between 20 and 40° (Fon et al., 1980). As soon as the Cobb angle exceeds the 95th percentile (>40°) it is considered as hyperkyphosis. Thoracic kyphosis tends to increase with age, implying that hyperkyphosis is more common in individuals aged 60 years or more, with a prevalence of 20–40 %, affecting women more frequently than men (Kado, 2007; Katzman et al., 2010; Kado et al., 2013; Katzman et al., 2014; Roghani et al., 2017). To date, the causes of hyperkyphosis are not yet elucidated but multiple factors have been identified: vertebral fractures, which are present in one third of the cases (Kado, 2007; Katzman et al., 2010; Kado et al., 2013; Katzman et al., 2014; Roghani et al., 2017; Schneider et al., 2004); degenerative disc disease (Kado, 2007; Katzman et al., 2010; Kado et al., 2013; Katzman et al., 2014; Roghani et al., 2017); postural changes with or without reduction of spinal extensor muscles strength (Kado, 2007; Katzman et al., 2010; Kado et al., 2013; Katzman et al., 2014; Roghani et al., 2017); genetic factors, with an estimated heritability of 54 % (Kado et al., 2013; Yau et al., 2016). Hyperkyphosis is associated with reduced physical performance resulting in a higher risk of falls and fractures, respiratory function impairment and a positive correlation between kyphosis angle and pain, culminating in decreased quality of life and higher overall mortality (Kado, 2007; Katzman et al., 2010; Kado et al., 2013; Katzman et al., 2014; Roghani et al., 2017; Schneider et al., 2004; Yau et al., 2016; Takahashi et al., 2005; Huang et al., 2005; Bruno et al., 2012).

As with thoracic kyphosis, a complete understanding of the mechanisms associated with aging remains challenging. Decline in mitochondrial function, linked to an increase in mitochondrial DNA (mtDNA) mutations, may be an important factor in aging and age-related pathologies, including kyphosis (Sun et al., 2016; Trifunovic et al., 2004; Kujoth et al., 2005; Trifunovic et al., 2005). Mice carrying a homozygous proof-reading deficient version of the mtDNA polymerase gene *Polg* are characterized by a reduced life span, with a maximum survival of 15 months, and all Polg mice display progeroid features such as kyphosis and sarcopenia that become apparent from the age of 9 months (Trifunovic et al., 2004; Kujoth et al., 2005; Shabalina et al., 2017). Increased apoptosis rates, but not reactive oxygen species production, between the age of 3 and 6 months in tissues with rapid cellular turnover has been identified as the pivotal mechanism underpinning the premature aging phenotype (Trifunovic et al., 2004; Kujoth et al., 2005; Trifunovic et al., 2005). Recent findings tend to challenge this mechanism, as increased mitochondrial hydrogen peroxide production was described in aging mutants (Logan et al., 2014) and treatment with a mitochondria-targeted antioxidant reduced kyphosis and other aging-associated traits (Shabalina et al., 2017).

We have previously demonstrated that homozygous *Polg*^*D257A*^ mice displayed osteopenia of articular subchondral bone and increased chondrocyte hypertrophy, but not accelerated development of knee osteoarthritis (Geurts et al., 2020). While osteoporosis has been suggested as causative for hyperkyphosis in prematurely aging mice (Trifunovic et al., 2004), spinal tissues have not been extensively characterized thus far. The purpose of our study was to compare vertebral bone structural parameters and intervertebral disc (IVD) morphology between mtDNA mutator mice and age-matched wild type littermates. We hypothesized that generalized or local vertebral bone loss leads to alterations in vertebral shape that predisposes to hyperkyphosis.

## 2 Materials and methods

### 2.1 Study design

This is a descriptive study using a convenience sample; therefore, no statistical methods were used to predetermine sample size. The experiments were not randomized and investigators were not blinded to allocation during experiments and outcome assessment (Geurts et al., 2020).

### 2.2 Mutator mice

MtDNA mutator mice were generated and phenotyped as described previously (Kujoth et al., 2005). Dissected thoracolumbar vertebral columns were obtained from *Polg*^*D257A*^ (*n* = 4, all male) and age-matched wild type littermates (*n* = 3, all male) aged 11, 12 and 14.5 months. The study protocol was approved by the Animal Care and Use Committee of the University of Wisconsin-Madison, USA. The experiments were performed in accordance with institutional guidelines and regulations.

### 2.3 Micro-computed tomography scanning and analysis

Thoracolumbar vertebral columns were fixed in formalin for 24 h and subsequently transferred to 70 % ethanol. Micro-computed tomography (μCT) sequences were performed using a benchtop micro-computed tomography scanner (Skyscan 1076, Bruker, Kontich, Belgium). The parameters were 59 kV, 167 μA, exposure time 50 ms, resolution of 19 μm, rotation step of 1° over 360°, and use of a 0.5 mm aluminum filter. The reconstruction of the sequence was performed with the Nrecon reconstruction software. Scans were reoriented using Dataviewer Version 1.5.3.4 (Bruker, Kontich, Belgium) and analyses were performed on CTAnalyzer Version 1.16.9.0+ (Bruker, Kontich, Belgium) and Surgimap Version 2.3.2.1 (Surgimap, Methuen, MA, USA).

Quantitative bone analysis was performed at T8-L4 vertebral levels using CTAnalyzer. To determine whether spine hyperkyphosis was associated with regional changes of trabecular and/or cortical bone, analyses were performed on two volumes of interest: the anterior and the posterior halves of vertebral bodies (Simpson et al., 2001; Banse et al., 2001; Gong et al., 2005; Wang et al., 2013). Trabecular bone was manually selected by interpolating several regions of interest and by applying a binary threshold, and its microarchitecture was assessed for the following measurements: Bone volume fraction (Tb.BV/TV [%]); Trabecular thickness (Tb.Th [μm]); Trabecular number (Tb.N [1/μm]); Trabecular separation (Tb.Sp [μm]). Cortical bone at these levels was also manually selected and evaluated for the following parameters: Cortical bone thickness (Ct.Th [μm]); Cortical bone mineral density (Ct.BMD [%]).

Analysis of vertebral shape was primarily performed with CTAnalyzer and included five measurements along the sagittal axis: anterior wall length; posterior wall length; caudal width; cranial width and central width (all expressed in [μm]). With these measurements, five ratios were calculated: Length ratio (anterior wall length/posterior wall length); Width ratio (caudal width/cranial width); Slenderness ratio (posterior length/caudal width); Thickness ratio (central width/caudal width) (Gorman et al., 2010). Vertebral sagittal wedge angle [°] was determined using Surgimap.

**Fig. 1:**
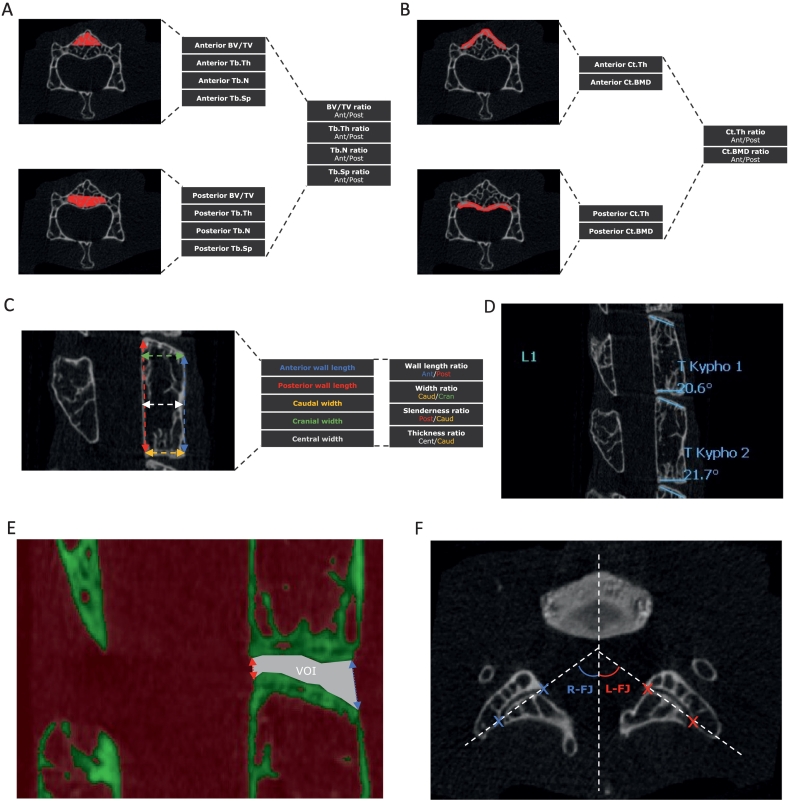
Description of VOI measurements and calculations used in the present study: trabecular bone parameters and anterior/posterior ratios (A); cortical bone parameters and anterior/posterior ratios (B); shape parameters and ratios (C); vertebral sagittal wedge angles (D); average, anterior and posterior IVD thickness (E); measurement of left and right facet joints angles according to Noren et al. (1991) (F).

The orientation of the T8-L4 facet joints (FJ) was assessed on transaxial μCT sequences with CTAnalyzer using the method described by Noren et al. (1991). Mean angles and FJ asymmetry (both in [°]) were used for direct comparison between groups. The methods for selecting volumes of interest and measurements are illustrated in Fig. 1.

### 2.4 IVD thickness measurements and histopathology

Average, anterior and posterior IVD thickness were determined on sagittal μCT sequences at T8-L4 levels using CTAnalyzer. (Fig. 1E). Lumbar vertebrae (L5-L6) were decalcified in EDTA for 2 weeks and embedded in paraffin. IVD gross morphology was assessed on 6 μm sagittal sections stained with Safranin-O/Fast Green.

### 2.5 Statistical analysis

Data are reported as absolute values and as ratios as previously mentioned. To compare regional vertebral differences between genotypes, morphometric data were expressed as anterior/posterior ratios. Statistical differences between groups were assessed using Student's *t-*test on GraphPad Prism 9.0 (GraphPad Software, Inc., La Jolla, USA). Pearson correlations were performed to determine associations between bone parameters of vertebrae and their subjacent IVD thickness. P values <0.05 were regarded as statistically significant.

## 3 Results

### 3.1 *Polg*^*D257A*^ mice display regional trabecular bone loss around the thoracolumbar junction and generalized cortical bone loss

**Fig. 2:**
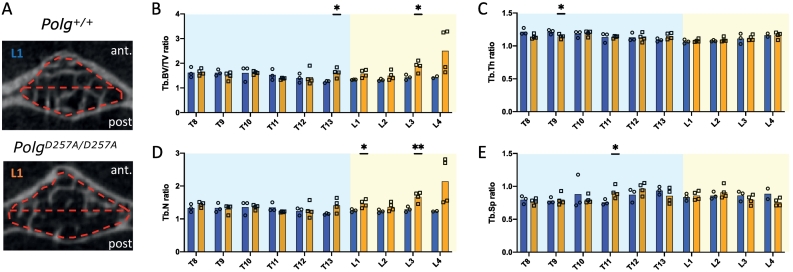
Comparative assessment of trabecular bone (Tb) from T8-L4 vertebrae with anterior/posterior ratios between wild type and mutant mice: two-dimensional transaxial μCT images of the L1 vertebral body with trabecular bone VOIs highlighted (A); anterior/posterior Tb.BV/TV ratio (B); anterior/posterior Tb.Th ratio (C); anterior/posterior Tb.N ratio (D); anterior/posterior Tb.Sp ratio (E). *p < 0.05, **p < 0.01 by Student's *t-*tests.

MtDNA mutator mice develop a premature aging phenotype characterized by osteoporosis, spine kyphosis, reduced body weight and loss of muscle mass (Trifunovic et al., 2004; Kujoth et al., 2005; Trifunovic et al., 2005). As vertebral fractures predispose to age-related hyperkyphosis in humans, we first determined bone structural parameters of anterior and posterior portions of vertebrae (Fig. 2A). In mutants, overall trabecular BV/TV, Tb.Th and Tb.N were significantly reduced at both the anterior and posterior portions of the T8-L4 vertebrae, with a 25 % and 30 % loss of Tb.BV/TV and a 15 % and 20 % loss of Tb.N in the anterior and posterior parts of vertebrae, respectively, but a balanced 15 % loss of Tb.Th in both parts (table in Supplementary Fig. 1). Global trabecular separation was not significantly modified between genotypes. When comparing the groups of mice according to each vertebra, the reduction in Tb.Th was discovered in most levels, but the decrease of BV/TV and Tb.N in the mutants was only significant at T9, T12 and T13 (Supplementary Fig. 1). *Polg*^*D257A*^ mice displayed a significant increase of anterior/posterior BV/TV and Tb.N ratios at the thoracolumbar junction (T13-L1) and at L3 (Fig. 2B & D). Yet, Tb.Th and Tb.Sp ratios remained essentially unchanged (Fig. 2C & E).

**Fig. 3:**
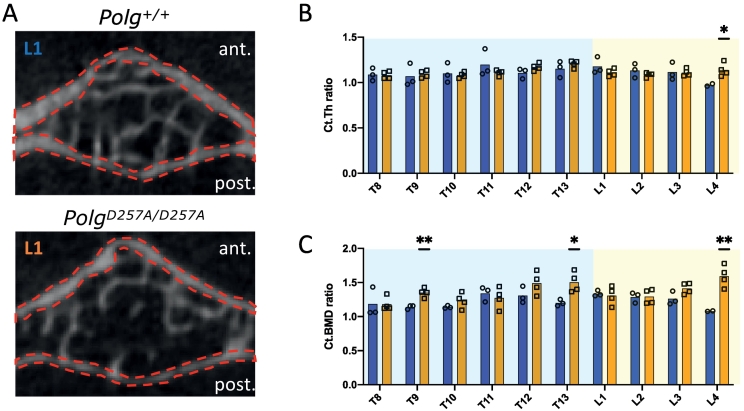
Comparative evaluation of cortical bone (Ct) from T8-L4 vertebrae with anterior/posterior ratios between wild type and mutant mice: two-dimensional transaxial μCT images of the L1 vertebral body with cortical bone VOIs highlighted (A); anterior/posterior Ct.Th ratio (B); anterior/posterior Ct.BMD ratio (C). *p < 0.05, **p < 0.01 by Student's *t-*tests.

Mutant mice displayed an overall ~25 % reduction of cortical bone in both the anterior part and posterior part of the vertebrae (Supplementary Fig. 2A), resulting in mostly no relevant difference in the anterior/posterior ratio of Ct.Th (Fig. 3A,B). However, the anterior/posterior Ct.BMD ratio increased significantly in the T9, T13 and L3 vertebrae (Fig. 3C), which also showed a reduction in BV/TV.

### 3.2 Vertebral shape remained unaltered despite trabecular and cortical bone loss

**Fig. 4:**
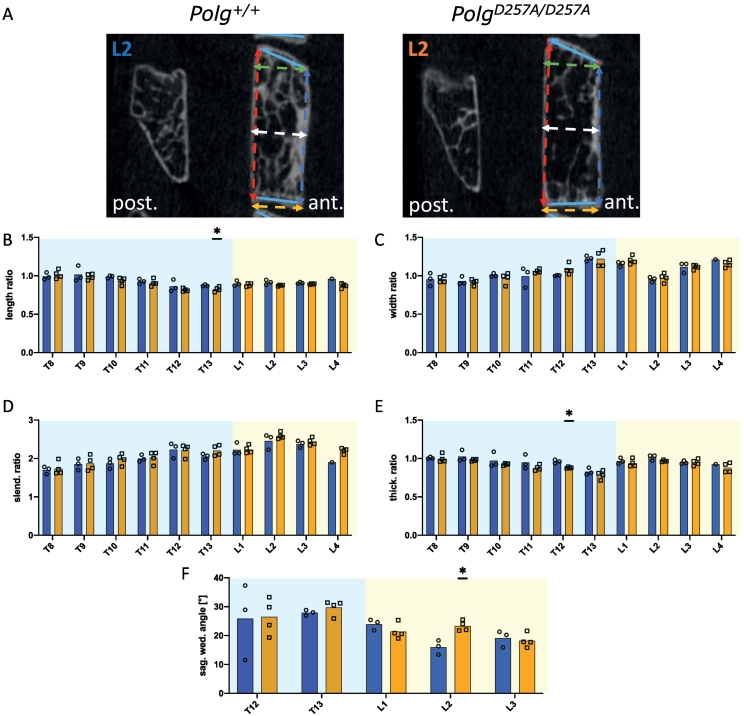
Two-dimensional sagittal μCT images of the L2 vertebrae showing shape parameters (A); Comparison of shape ratios in wild type and mutant mice: wall length ratio (anterior wall length/posterior wall length) (B); width ratio (caudal width/cranial width) (C); slenderness ratio (posterior wall length/caudal width) (D); thickness ratio (central width/caudal width) (E); sagittal wedge angle of the thoracolumbar vertebrae (F). *p < 0.05 by Student's *t-*tests.

Next, to determine whether hyperkyphosis in homozygous mice is associated with changes in vertebral shape, we determined several shape parameters measurements on sagittal μCT (Gorman et al., 2010). Wall lengths, sagittal endplate widths and central vertebral widths revealed no significant difference between genotypes for most vertebrae (Fig. 4A–D). There was no evidence of vertebral fracture. Further examination of thoracolumbar vertebrae wedge angles did not show any significant difference, despite a 30 % increase of the wedge angle at L2 (Fig. 4F).

**Fig. 5:**
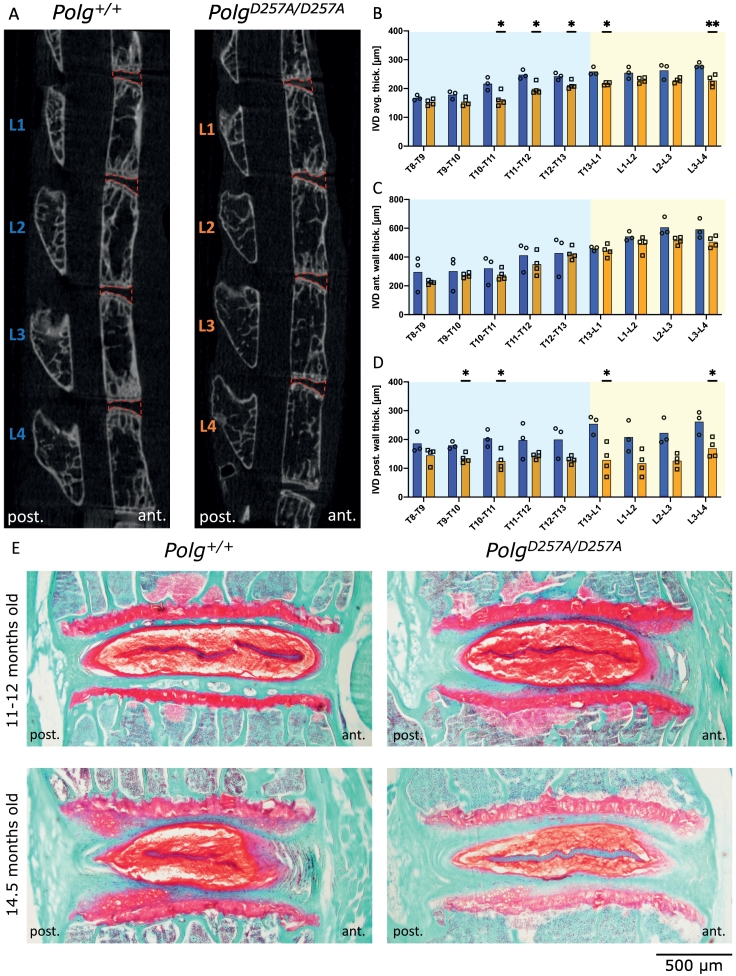
Two-dimensional mid-sagittal μCT images of thoracolumbar vertebrae with IVDs VOIs highlighted (A); T8-T9 to L3-L4 IVDs average thickness [μm] (B); T8-T9 to L3-L4 IVDs posterior thickness [μm] (C); T8-T9 to L3-L4 IVDs anterior thickness [μm] (D); mid-sagittal sections of lumbar IVDs (L5-L6) with Safranin-O/Fast Green/Haematoxylin staining for each genotype (E). *p < 0.05, **p < 0.01 by Student's *t-*tests.

**Fig. 6:**
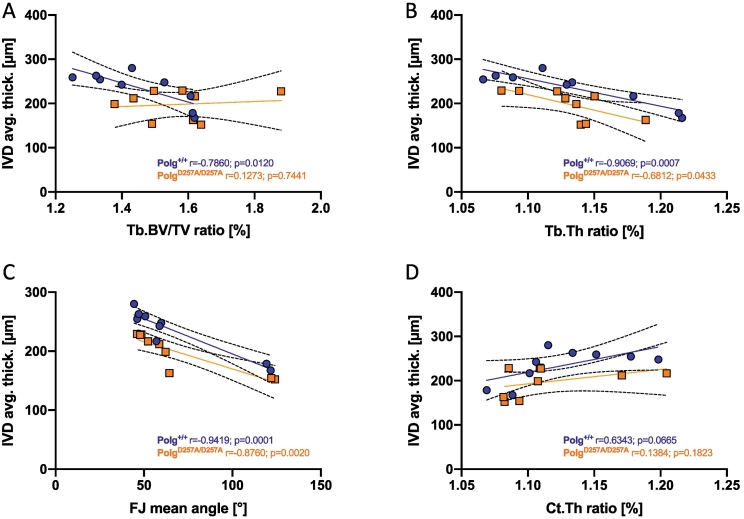
Pearson correlation analysis of mean values of anterior/posterior ratios from vertebrae (T8-L4) and their subjacent IVD thickness, in wild type (blue circles) and homozygous (orange squares) mice: anterior/posterior Tb.BV/TV ratio and IVD thickness (A); anterior/posterior Tb.Th ratio and IVD thickness (B); anterior/posterior Ct.Th ratio and IVD thickness (C); FJ mean angle and IVD thickness (D). Regression lines with 95 % confidence intervals, as well as correlation indexes and respective p values are displayed for wild types and homozygotes on each graph with corresponding colors.

### 3.3 Homozygous mice display reduced posterior IVD thickness without histopathological features

Next, we measured average, anterior and posterior IVD thickness of both genotypes using μCT (Fig. 5A). IVDs of *Polg*^*D257A*^ mice were overall 15 % thinner and respective measurements of the anterior and posterior disc walls revealed that this reduction in IVD thickness is primarily related to a narrowing of the posterior intervertebral space at the thoracic and lumbar levels (Fig. 5B–D, Supplementary Fig. 3). Histology of L5-L6 IVDs revealed no major differences between genotypes. Homozygous mtDNA mutator and wild type mice showed a proteoglycan-rich nucleus pulposus and an intact lamellar structured annulus fibrosis without evidence of fissure and the interface between the two structures was well delineated (Fig. 5E).

### 3.4 No significant difference in the transaxial facet joint orientation

Previous studies suggest that there may be a relationship between degenerative disc disease and variations in FJ alignment and other degenerative spinal disorders, but this association is still debated in the literature (Noren et al., 1991; Liu et al., 2017; Garg and Aggarwal, 2021). Acting instead as an outcome of interest, we sought to determine whether spinal hyperkyphosis was accompanied by changes in FJ orientation. FJ angles were not statistically different between mutant and wild type littermates (*Polg*^*D257A*^: 69.57 ± 30.99°; WT: 67.2 ± 30.71°. p = 0.8728). While there was larger variation in FJ asymmetry in mtDNA mutator mice, differences between genotypes were not statistically significant (*Polg*^*D257A*^: 5.361 ± 2.244°; WT: 3.622 ± 1.744°. p = 0.0851) (Supplementary Fig. 4).

### 3.5 IVD thickness is negatively correlated to anterior/posterior bone parameter ratios and facet joint coronalization

Pearson correlation analysis revealed that IVD thickness was negatively correlated with the anterior/posterior ratio of BV/TV and Tb.Th (Fig. 6A,B), but positively correlated with the anterior/posterior ratio of Ct.Th (Fig. 6D). IVD thickness was also negatively associated with more coronally aligned FJ (Fig. 6C). Finally, analyses of anterior and posterior portions revealed that IVD thickness and bone structural parameters were significantly associated in the prior, but not the latter (Supplementary Fig. 5). These results indicate that a larger difference in anterior/posterior bone parameters could correspond with reduced IVD thickness.

## 4 Discussion

This study described for the first time the bone and cartilage features of spinal hyperkyphosis associated with mtDNA mutations and mitochondrial dysfunction. MtDNA mutator mice were associated with diffuse trabecular bone loss with posterior regions being more affected at the lower thoracic and lumbar levels, but overall diffuse cortical bone loss. Nevertheless, this pattern of osteopenia was insufficient to induce a substantial change in vertebral shape. Disc space narrowing was also greater in homozygous mutants, but further histological analysis could not confirm increased IVD degeneration in these subjects. The orientation of FJ on the transaxial plane did not differ significantly, but the mutants appeared to have more coronally aligned and greater variability in FJ asymmetry than their wild type counterparts. Lastly, IVD thickness was negatively correlated with Tb.Th ratio and FJ angles.

High bone turnover rates are associated with osteoporosis when osteoblasts cannot compensate for osteoclast activity, particularly in the setting of estrogen deficiency and Polg mice are known to display gonadal atrophy as early as 12 weeks of age (Trifunovic et al., 2004; Kujoth et al., 2005; Weitzmann, 2006). However, bone remodelling may be protective against spinal hyperkyphosis if it is characterized by a balanced increase in bone formation and resorption (McDaniels-Davidson et al., 2016 Dec). Previous analyses in our laboratory, using the same mouse model, detected osteopenia in mutants with an increased rate of osteocyte apoptosis and an elevated number of osteoclasts (Geurts et al., 2020). Dobson & al. demonstrated similar histomorphometric results in lumbar vertebrae, with mutants displaying a 10–20 % reduction in Tb.BV/TV at 11 months of age, and found that *Polg*^*D257A*^ mice exhibited impaired osteogenic differentiation of mesenchymal stem cells, resulting in fewer mature osteoblasts and progressively decreased osteoformation and matrix mineralization (Dobson et al., 2020). Scheuren *et al* detected similar evolution of trabecular bone in mtDNA mutator mice, with a progressive decrease in BV/TV and Tb.Th, but no significant difference in Tb.N and Tb.Sp (Scheuren et al., 2020). A central part of this study was devoted to the existence of regional variations in the microarchitecture of the trabecular bone of vertebral bodies. Banse *et al* demonstrated that inhomogeneity exists in human vertebral trabecular bone, with posterior parts displaying up to 20 % higher values of Tb.BV/TV (Banse et al., 2001), which appeared to be the opposite in mice, with higher Tb.BV/TV, Tb.Th and Tb.N values in anterior parts. This difference can be partially attributed to the fact that humans are characterized by a bipodal posture that increases loads on the neural arch and mice are quadrupeds with a horizontal orientation of the spine (Smit, 2002). Gong *et al* analyzed human lumbar vertebrae and demonstrated that aging was associated with different degrees of reduction in histomorphometric parameters depending on location, with greater decrements in Tb.BV/TV in the peripheral regions than in central vertebral regions (Gong et al., 2005). Other authors have linked microarchitecture disturbances to disc degeneration, reporting an increase in bone fraction in response to advancing IVD disease and increased risk of subsequent vertebral fracture (Simpson et al., 2001; Wang et al., 2013; Tzermiadianos et al., 2008; Kague et al., 2021). Hyperkyphosis is also recognized as an independent risk factor for vertebral fractures as a result of an anterior shift of the body weight, which increases the load and torque on the anterior part of vertebral bodies (Huang et al., 2005; Bruno et al., 2012; Briggs et al., 2007). The explanation for the exacerbated regional pattern of bone loss in mutants at the thoracolumbar junction is certainly multifactorial, related to disturbances in mitochondrial function and mechanical disruption at the discovertebral junction due to altered sagittal balance, but is also likely to involve the interface between the rigid thoracic portion of the spine to the more mobile lumbar vertebrae, which is known to be a region of high mechanical stress (Wood et al., 2014). However, the results suggested that these changes in bone histomorphometry were insufficient to significantly alter the shape of the vertebrae, which may partly be related to the higher trabecular Tb.BV/TV values observed in the anterior parts of the mice vertebrae. Furthermore, wedging of the thoracolumbar hinge vertebrae has been reported in asymptomatic individuals and is not consistently suggestive of a vertebral fracture (Matsumoto et al., 2011). Thus, trabecular bone manifestations may be of minor importance to the onset of hyperkyphosis in *Polg*^*D257A*^ subjects and are rather consequences of the premature aging phenotype and biomechanical changes caused by spinal deformity since no evidence of vertebral fracture was detected. The importance of the latter in humans should not be underestimated, as they are present in one third of cases and are responsible for kyphotic spines with dramatic angulations with each fracture being able to increase the Cobb angle up to 3.8 degrees (Kado, 2007; Kado et al., 2013). Conversely, kyphotic spines can also occur in the absence of any endplate anomaly (Schneider et al., 2004).

Aging is associated with changes in IVDs, which occur as early as the second decade, such as maturation of chondrocytes in the NP and increased matrix turnover and fibrosis, caused by an increased production of inflammatory cytokines and collagen type 1, but decreased levels of collagen type 2 and proteoglycans (Antoniou et al., 1996; Urban and Roberts, 2003), modulated by the mechanical and biological status of adjacent structures such as the AF and vertebral endplates (Simpson et al., 2001; Wang et al., 2013; Tzermiadianos et al., 2008; Kague et al., 2021). Moreover, recent data provided evidence that endplate osteoporosis was not protective against IVD degeneration, and a positive correlation also associated high BMD to IVD disease (Kague et al., 2021), which could partly explain our negative associations between increased Tb.BV/TV or Tb.Th and IVD thickness. As shown in our previous study, *Polg*^*D257A*^ subjects were characterized by reduced proteoglycan content in the knee joints, but no difference in chondrocyte apoptosis rates in articular and growth plate cartilage, in addition to an increased number of hypertrophied chondrocytes in calcified cartilage (Geurts et al., 2020). Loss of proteoglycans may be involved in the decrease in average IVD thickness observed in homozygous mutants, through dehydration of NPs (Antoniou et al., 1996; Urban and Roberts, 2003). The explanation for the posterior rather than the anterior thinning of the IVDs, mainly observed at the lumbar vertebrae, may be related to a shift of compression to the neural arch at this level of the spine, as hyperkyphosis is sometimes associated with compensatory hyperlordosis and FJ degeneration (Roussouly and Pinheiro-Franco, 2011; Oda et al., 1999). However, the trabecular bone fraction was also greater in the anterior part of the lumbar vertebrae in mutants, indirectly suggesting that stress on the IVDs and endplates was greater at this location, making compensatory lumbar hyperlordosis less likely than loss of lumbar lordosis in the mutants (Simpson et al., 2001; Wang et al., 2013; Roussouly and Pinheiro-Franco, 2011; Pearson and Lieberman, 2004; Barrey et al., 2013). It is conceivable that these anterior constraints force the NP posteriorly and that kyphosis increases stretch stresses on the posterior AF, resulting in degeneration and collapse of the posterior AF at the thoracic and thoracolumbar levels (Liu et al., 2014). Yet, other signs of more advanced degenerative IVD disease could not be identified in homozygous mutants, which is probably related to the rarity of natural development of IVD disease in mouse models, suggested indirectly by the need to induce it experimentally or surgically in mice (Singh et al., 2005; Daly et al., 2016), but also to the short life expectancy of mutants. The results also showed an inverse association between FJ coronalization and IVD thickness, but the evidence linking FJ orientation and asymmetry to IVD degeneration in not unanimous, with several authors suggesting a statistically significant association of the two processes, whereas others have not (Noren et al., 1991; Liu et al., 2017; Garg and Aggarwal, 2021).

We acknowledge several limitations to our study. Statistical power was reduced by the relatively small sample size and did not enable us to verify effect sizes smaller than 20 % relative change. Second, partial occupancy effects at the given microCT resolution might significantly influence the measurements of trabecular microarchitecture. A major limitation of the present study is that analysis of the paraspinal muscles was impossible because the latter had been previously removed from mouse carcasses. Sarcopenia and alterations in paraspinous muscle composition and strength are risk factor of spinal hyperkyphosis as well as other spine disorders (Kado, 2007; Katzman et al., 2010; Kado et al., 2013; Katzman et al., 2014; Roghani et al., 2017; Hiona et al., 2010; Hiyama et al., 2018), and it was previously proven that an inverse correlation exists between paraspinal muscle area and kyphosis angle (Katzman et al., 2014; Yau et al., 2016). Quadriceps and gastrocnemius muscles from *Polg*^*D257A*^ subjects have been described previously and increasing levels of mtDNA mutations resulted with significant muscle atrophy through increased apoptosis and downregulation of aerobic cellular processes (Hiona et al., 2010). However, sarcopenia appeared to differ by muscle: in mutants, the quadriceps muscle mass was reduced by 40 % and the gastrocnemius muscle was only reduced by 24.5 % compared to wild type mice. It is also reported that stimulation of residual oxidative activity in mutants through endurance exercise significantly improved skeletal muscle mass and function when compared to sedentary mutants (Safdar et al., 2011), without having any substantial effect on bone (Dobson et al., 2020) but the effect of physical activity on kyphosis and paraspinal muscles were not specifically evaluated. To our knowledge, no study has attempted to characterize the extent of atrophy of the muscles responsible for spinal extension and flexion in this model of aging, implying that the role of muscle atrophy in this context can only be speculated. Based on previous evidence, it would be conceivable that the degree of muscle mass reduction in Polg mutants differs between the muscles responsible for extension and flexion of the spine, with atrophy being more pronounced in paraspinal muscles. The use of methods similar to the previously cited studies (*i.e.*, muscle imaging and biological techniques) to better characterize the respective alterations in erectors and flexors of the spine would allow to further clarify the involvement of muscles in hyperkyphosis and measure the impact of lifestyle or conservative interventions in preventing or decreasing the consequences of spinal hyperkyphosis.

## 5 Conclusions

Ultimately, thoracolumbar hyperkyphosis observed in *Polg*^*D257A*^ mice is accompanied by a pattern of regional loss of bone quality whose direct involvement may be of minor importance for the occurrence of this deformity. However, the decrease in trabecular and cortical bone quality might be an additional risk of worsening of this deformity through the subsequent development of vertebral fractures. Furthermore, vertebral bone status as well as FJ coronalization were inversely correlated with IVD thickness, which may be more relevant to the emergence of hyperkyphosis. The role of paraspinal and trunk musculature alterations is very likely, but further evaluation is required to determine its relative contribution to hyperkyphosis in the context of aging and somatically acquired mtDNA mutations.

The following are the supplementary data related to this article:

Supplementary Fig. 1 Comparative assessment of anterior (left column) and posterior (right column) trabecular bone parameters between wild type (blue bars) and mutant mice (orange bars): Tb.BV/TV [%] (A); Tb.Th [μm] (B); Tb.N [1/μm] (C); Tb.Sp [μm] (D). Supplementary table with descriptive results from T8-L4 comparison (E). *p *<* *0.05,* **p < 0.01, ***p < 0.001 by Student's *t-*tests. Supplementary Fig. 1

Supplementary Fig. 2 Comparative evaluation of anterior (left column) and posterior (right column) cortical bone parameters between wild type (blue bars) and mutant mice (orange bars): Ct.Th [μm] (A); Ct.BMD [%] (B). Supplementary table with descriptive results from T8-L4 comparison (C). *p < 0.05, **p < 0.01, ***p < 0.001 by Student's *t-*tests. Supplementary Fig. 2

Supplementary Fig. 3 Comparative evaluation of average, anterior and posterior IVD thickness between wild type and mutant mice: Supplementary table with descriptive results from T8-L4 comparison with respective p values by Student's *t-*tests. Supplementary Fig. 3

Supplementary Fig. 4 Comparative analysis of the transaxial orientation of the FJ at the T8-L4 spinal levels of wild type and mutant mice: two-dimensional transaxial μCT images of L1-L2 FJ with angle measurement according to Noren et al. (1991) (A); mean FJ angle (B); mean difference between right and left FJ angles (C). Data are expressed as fold over wild type (blue line), *p < 0.05 by Student's *t-*tests. Supplementary Fig. 4

Supplementary Fig. 5 Pearson correlation analysis of mean values of bone parameters in the anterior (upper row) and posterior (lower row) portions of vertebrae (T8-L4) and their subjacent IVD anterior and posterior thickness, in wild type (blue circles) and homozygous (orange squares) mice: Tb.BV/TV and IVD thickness (A); Tb.Th and IVD thickness (B); Ct.Th and IVD thickness (C). Regression lines with 95 % confidence intervals, as well as correlation indexes and respective p values are displayed for wild types and homozygotes on each graph with corresponding colors. Supplementary Fig. 5

Thoracic spine. Image 1

Lumbar spine. Image 2

## CRediT authorship contribution statement

**Olivier Roessinger:** Conceptualization, Methodology, Formal analysis, Investigation, Writing – original draft, Visualization. **Thomas Hügle:** Conceptualization, Methodology, Writing – review & editing, Visualization, Supervision. **Ulrich A. Walker:** Conceptualization, Resources, Writing – review & editing. **Jeroen Geurts:** Conceptualization, Methodology, Formal analysis, Investigation, Writing – review & editing, Supervision, Project administration, Funding acquisition.

## Declaration of competing interest

The authors declare no competing interests.

## References

[bb0005] Antoniou J., Steffen T., Nelson F., Winterbottom N., Hollander A.P., Poole R.A. (1996). The human lumbar intervertebral disc: evidence for changes in the biosynthesis and denaturation of the extracellular matrix with growth, maturation, ageing, and degeneration. J. Clin. Invest..

[bb0010] Banse X., Devogelaer J.P., Munting E., Delloye C., Cornu O., Grynpas M. (2001). Inhomogeneity of human vertebral cancellous bone: systematic density and structure patterns inside the vertebral body. Bone.

[bb0015] Barrey C., Roussouly P., Le Huec J.C., D’Acunzi G., Perrin G. (2013). Compensatory mechanisms contributing to keep the sagittal balance of the spine. Eur. Spine J..

[bb0020] Briggs A.M., van Dieën J.H., Wrigley T.V., Greig A.M., Phillips B., Lo S.K. (2007). Thoracic kyphosis affects spinal loads and trunk muscle force. Phys. Ther..

[bb0025] Bruno A.G., Anderson D.E., D’Agostino J., Bouxsein M.L. (2012). The effect of thoracic kyphosis and sagittal plane alignment on vertebral compressive loading. J. Bone Miner. Res..

[bb0030] Daly C., Ghosh P., Jenkin G., Oehme D., Goldschlager T. (2016). A review of animal models of intervertebral disc degeneration: pathophysiology, regeneration, and translation to the clinic. Biomed. Res. Int..

[bb0035] Dobson P.F., Dennis E.P., Hipps D., Reeve A., Laude A., Bradshaw C. (2020). Mitochondrial dysfunction impairs osteogenesis, increases osteoclast activity, and accelerates age related bone loss. Sci. Rep..

[bb0040] Fon G., Pitt M., Thies A. (1980). Thoracic kyphosis: range in normal subjects. Am. J. Roentgenol..

[bb0045] Garg K., Aggarwal A. (2021). Facet tropism in lumbar spine and cervical spine: a systematic review and meta-analysis. World Neurosurg..

[bb0050] Geurts J., Nasi S., Distel P., Müller-Gerbl M., Prolla T.A., Kujoth G.C. (2020). Prematurely aging mitochondrial DNA mutator mice display subchondral osteopenia and chondrocyte hypertrophy without further osteoarthritis features. Sci. Rep..

[bb0055] Gong H., Zhang M., Yeung H.Y., Qin L. (2005). Regional variations in microstructural properties of vertebral trabeculae with aging. J. Bone Miner. Metab..

[bb0060] Gorman K.F., Handrigan G.R., Jin G., Wallis R., Breden F. (2010). Structural and micro-anatomical changes in vertebrae associated with idiopathic-type spinal curvature in the curveback guppy model. Scoliosis.

[bb0065] Hiona A., Sanz A., Kujoth G.C., Pamplona R., Seo A.Y., Hofer T. (2010). Mitochondrial DNA mutations induce mitochondrial dysfunction, apoptosis and sarcopenia in skeletal muscle of mitochondrial DNA mutator mice. PLoS One.

[bb0070] Hiyama A., Katoh H., Sakai D., Sato M., Tanaka M., Nukaga T. (2018). Correlation analysis of sagittal alignment and skeletal muscle mass in patients with spinal degenerative disease. Sci. Rep..

[bb0075] Huang M.H., Barrett-Connor E., Greendale G.A., Kado D.M. (2005). Hyperkyphotic posture and risk of future osteoporotic fractures: the rancho bernardo study. J. Bone Miner. Res..

[bb0080] Kado D.M. (2007). Narrative review: hyperkyphosis in older persons. Ann. Intern. Med..

[bb0085] Kado D.M., Huang M.H., Karlamangla A.S., Cawthon P., Katzman W., Hillier T.A. (2013). Factors associated with kyphosis progression in older women: 15 years’ experience in the study of osteoporotic fractures. J. Bone Miner. Res..

[bb0090] Kague E., Turci F., Newman E., Yang Y., Brown K.R., Aglan M.S. (2021). 3D assessment of intervertebral disc degeneration in zebrafish identifies changes in bone density that prime disc disease. Bone Res..

[bb0095] Katzman W.B., Wanek L., Shepherd J.A., Sellmeyer D.E. (2010). Age-related hyperkyphosis: its causes, consequences, and management. J. Orthop. Sports Phys. Ther..

[bb0100] Katzman W.B., Miller-Martinez D., Marshall L.M., Lane N.E., Kado D.M. (2014). Kyphosis and paraspinal muscle composition in older men: a cross-sectional study for the osteoporotic fractures in men (MrOS) research group. BMC Musculoskelet. Disord..

[bb0105] Kujoth G.C., Hiona A., Pugh T.D., Someya S., Panzer K., Wohlgemuth S.E., Hofer T., Seo A.Y., Sullivan R., Jobling W.A., Morrow J.D., Van Remmen H., Sedivy J.M., Yamasoba T., Tanokura M., Weindruch R., Leeuwenburgh C., Prolla T.A. (2005). Mitochondrial DNA mutations, oxidative stress, and apoptosis in mammalian aging. Science.

[bb0110] Liu N., Chen Z., Qi Q., Shi Z. (2014). The relationship of symptomatic thoracolumbar disc herniation and Scheuermann's disease. Eur. Spine J..

[bb0115] Liu Z., Duan Y., Rong X., Wang B., Chen H., Liu H. (2017). Variation of facet joint orientation and tropism in lumbar degenerative spondylolisthesis and disc herniation at L4–L5: a systematic review and meta-analysis. Clin. Neurol. Neurosurg..

[bb0120] Logan A., Shabalina I.G., Prime T.A., Rogatti S., Kalinovich A.V., Hartley R.C. (2014). In vivo levels of mitochondrial hydrogen peroxide increase with age in mt DNA mutator mice. Aging Cell.

[bb0125] Matsumoto M., Okada E., Kaneko Y., Ichihara D., Watanabe K., Chiba K. (2011). Wedging of vertebral bodies at the thoracolumbar junction in asymptomatic healthy subjects on magnetic resonance imaging. Surg. Radiol. Anat..

[bb0130] McDaniels-Davidson C.R., Kritz-Silverstein D., Huang M.H., Laughlin G.A., Johnson S., Haapalahti J. (2016 Dec). The association between bone turnover markers and kyphosis in community-dwelling older adults. Bone Rep..

[bb0135] Noren R., Trafimow J., Andersson G.B.J., Huckman M.S. (1991). The role of facet joint tropism and facet angle in disc degeneration. Spine.

[bb0140] Oda I., Cunningham B.W., Buckley R.A., Goebel M.J., Haggerty C.J., Orbegoso C.M. (1999). Does spinal kyphotic deformity influence the biomechanical characteristics of the adjacent motion segments? An in vivo animal model. Spine.

[bb0145] Pearson O.M., Lieberman D.E. (2004). The aging of Wolff's ?law?: ontogeny and responses to mechanical loading in cortical bone. Am. J. Phys. Anthropol..

[bb0150] Roghani T., Zavieh M.K., Manshadi F.D., King N., Katzman W. (2017). Age-related hyperkyphosis: update of its potential causes and clinical impacts—narrative review. Aging Clin. Exp. Res..

[bb0155] Roussouly P., Pinheiro-Franco J.L. (2011). Biomechanical analysis of the spino-pelvic organization and adaptation in pathology. Eur. Spine J..

[bb0160] Safdar A., Bourgeois J.M., Ogborn D.I., Little J.P., Hettinga B.P., Akhtar M. (2011). Endurance exercise rescues progeroid aging and induces systemic mitochondrial rejuvenation in mtDNA mutator mice. Proc. Natl. Acad. Sci..

[bb0165] Scheuren A.C., D’Hulst G., Kuhn G.A., Masschelein E., Wehrle E., De Bock K. (2020). Hallmarks of frailty and osteosarcopenia in prematurely aged PolgA(D257A/D257A) mice. J. Cachexia. Sarcopenia Muscle.

[bb0170] Schneider D.L., von Mühlen D., Barrett-Connor E., Sartoris D.J. (2004). Kyphosis does not equal vertebral fractures: the rancho bernardo study. J. Rheumatol..

[bb0175] Shabalina I.G., Vyssokikh M.Yu., Gibanova N., Csikasz R.I., Edgar D., Hallden-Waldemarson A. (2017). Improved health-span and lifespan in mtDNA mutator mice treated with the mitochondrially targeted antioxidant SkQ1. Aging.

[bb0180] Simpson E.K., Parkinson I.H., Manthey B., Fazzalari N.L. (2001). Intervertebral disc disorganization is related to trabecular bone architecture in the lumbar spine. J. Bone Miner. Res. Off. J. Am. Soc. Bone Miner. Res..

[bb0185] Singh K., Masuda K., An H.S. (2005). Animal models for human disc degeneration. Spine J..

[bb0190] Smit T.H. (2002). The use of a quadruped as an in vivo model for the study of the spine – biomechanical considerations. Eur. Spine J..

[bb0195] Sun N., Youle R.J., Finkel T. (2016). The mitochondrial basis of aging. Mol. Cell.

[bb0200] Takahashi T., Ishida K., Hirose D., Nagano Y., Okumiya K., Nishinaga M. (2005). Trunk deformity is associated with a reduction in outdoor activities of daily living and life satisfaction in community-dwelling older people. Osteoporos. Int..

[bb0205] Trifunovic A., Wredenberg A., Falkenberg M., Spelbrink J.N., Rovio A.T., Bruder C.E. (2004). Premature ageing in mice expressing defective mitochondrial DNA polymerase. Nature.

[bb0210] Trifunovic A., Hansson A., Wredenberg A., Rovio A.T., Dufour E., Khvorostov I. (2005). Somatic mtDNA mutations cause aging phenotypes without affecting reactive oxygen species production. Proc. Natl. Acad. Sci..

[bb0215] Tzermiadianos M.N., Renner S.M., Phillips F.M., Hadjipavlou A.G., Zindrick M.R., Havey R.M. (2008). Altered disc pressure profile after an osteoporotic vertebral fracture is a risk factor for adjacent vertebral body fracture. Eur. Spine J..

[bb0220] Urban J.P.G., Roberts S. (2003). Degeneration of the intervertebral disc. Arthritis Res Ther..

[bb0225] Wang Y., Owoc J.S., Boyd S.K., Videman T., Battié M.C. (2013). Regional variations in trabecular architecture of the lumbar vertebra: associations with age, disc degeneration and disc space narrowing. Bone.

[bb0230] Weitzmann M.N. (2006). Estrogen deficiency and bone loss: an inflammatory tale. J. Clin. Invest..

[bb0235] Wood K.B., Li W., Lebl D.S., Ploumis A. (2014). Management of thoracolumbar spine fractures. Spine J..

[bb0240] Yau M.S., Demissie S., Zhou Y., Anderson D.E., Lorbergs A.L., Kiel D.P. (2016). Heritability of thoracic spine curvature and genetic correlations with other spine traits: the Framingham study: thoracic curvature heritability, correlations with other spine traits. J. Bone Miner. Res..

