# Supplementary material for: Polg mtDNA mutator mice reveal limited involvement of vertebral bone loss in premature aging-related thoracolumbar hyperkyphosis
Source: Bone Rep. 2022 Aug 30;17:101618. doi: 10.1016/j.bonr.2022.101618 (PMC9479024; doi:10.1016/j.bonr.2022.101618)
Supplement: Supplementary Fig. 1 — Comparative assessment of anterior (left column) and posterior (right column) trabecular bone parameters between wild type (blue bars) and mutant mice (orange bars): Tb.BV/TV [%] (A); Tb.Th [μm] (B); Tb.N [1/μm] (C); Tb.Sp [μm] (D). Supplementary table with descriptive results from T8-L4 comparison (E). *p < 0.05, **p < 0.01, ***p < 0.001 by Student's t-tests. [file mmc1.pdf]

Supp Fig 1

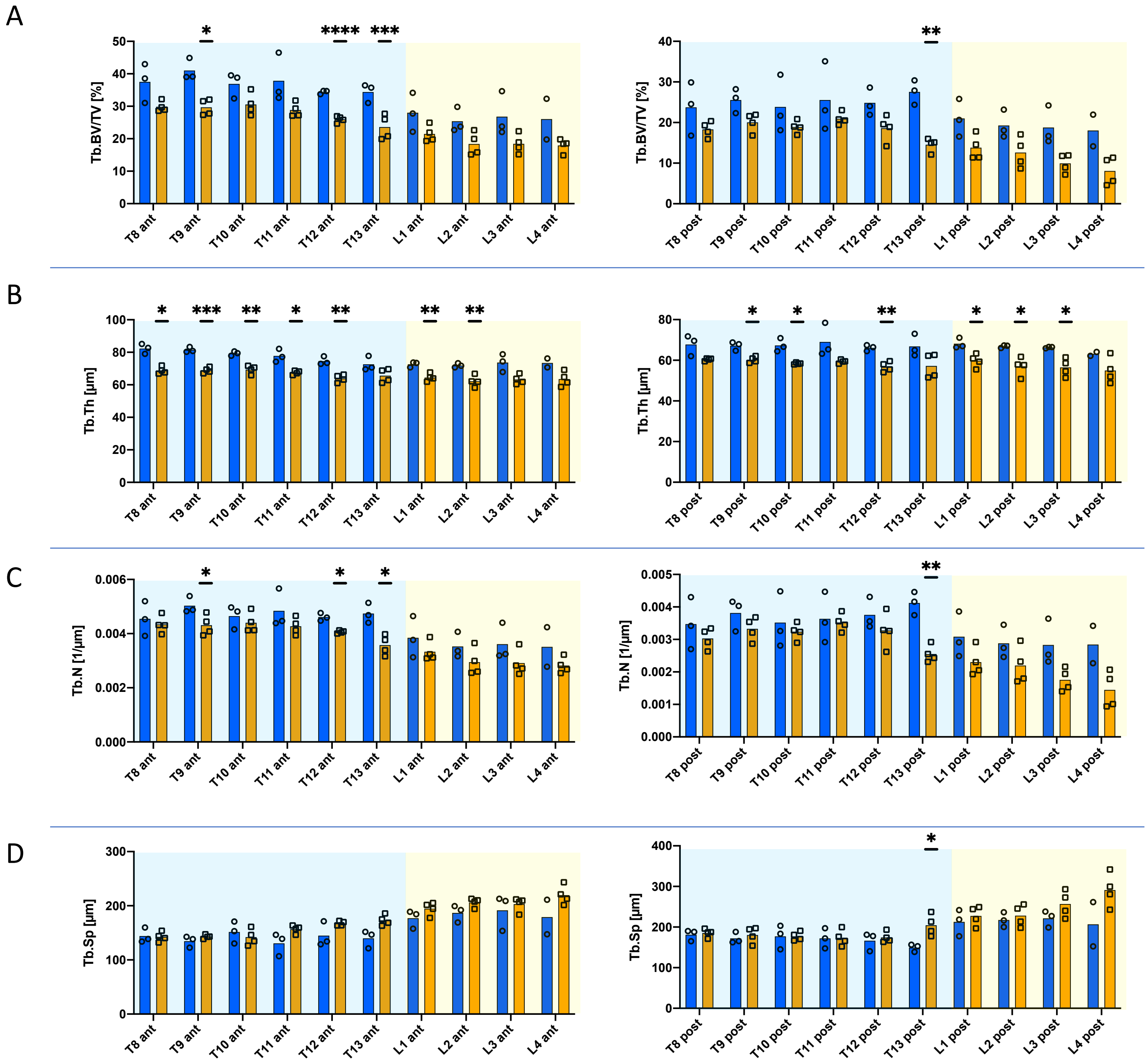

E

		anterior (T8-L4)				***posterior (T8-L4)***			
		Mean	***SD***	***P value***	***t***	***Mean***	***SD***	***P value***	***t***
Tb.BV/TV [%]	*WT*	32.830	5.741	0.003	3.419	22.820	3.302	0.001	4.133
	Polg D257A	24.480	5.177			15.590	4.443		
Tb.Th [µm]	*WT*	75.980	3.889	<0.0001	6.834	66.860	1.569	<0.0001	11.480
	Polg D257A	65.780	2.671			58.070	1.844		
Tb.N [1/µm]	*WT*	0.00429	0.00060	0.047	2.130	0.00340	0.00046	0.014	2.707
	Polg D257A	0.00370	0.00065			0.00267	0.00072		
Tb.Sp [µm]	*WT*	158.100	23.130	0.172	1.424	187.700	24.910	0.155	1.483
	Polg D257A	174.800	29.240			209.900	40.270		
