# Supplementary figures and images for: Polg mtDNA mutator mice reveal limited involvement of vertebral bone loss in premature aging-related thoracolumbar hyperkyphosis
Source: Bone Rep. 2022 Aug 30;17:101618. doi: 10.1016/j.bonr.2022.101618 (PMC9479024; doi:10.1016/j.bonr.2022.101618)

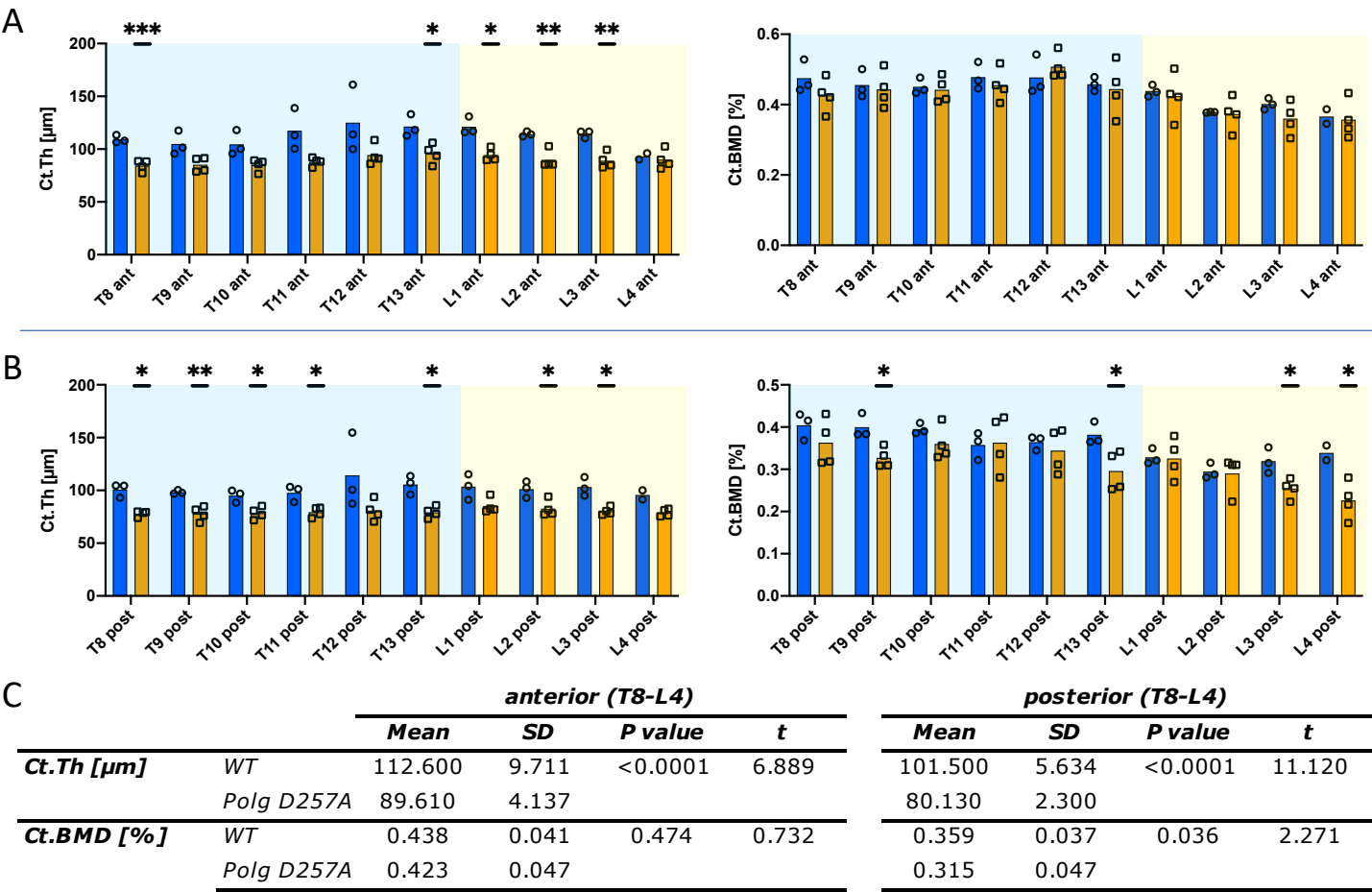

Supplement: Supplementary Fig. 2 — Comparative evaluation of anterior (left column) and posterior (right column) cortical bone parameters between wild type (blue bars) and mutant mice (orange bars): Ct.Th [μm] (A); Ct.BMD [%] (B). Supplementary table with descriptive results from T8-L4 comparison (C). *p < 0.05, **p < 0.01, ***p < 0.001 by Student's t-tests. [file mmc2.pdf]

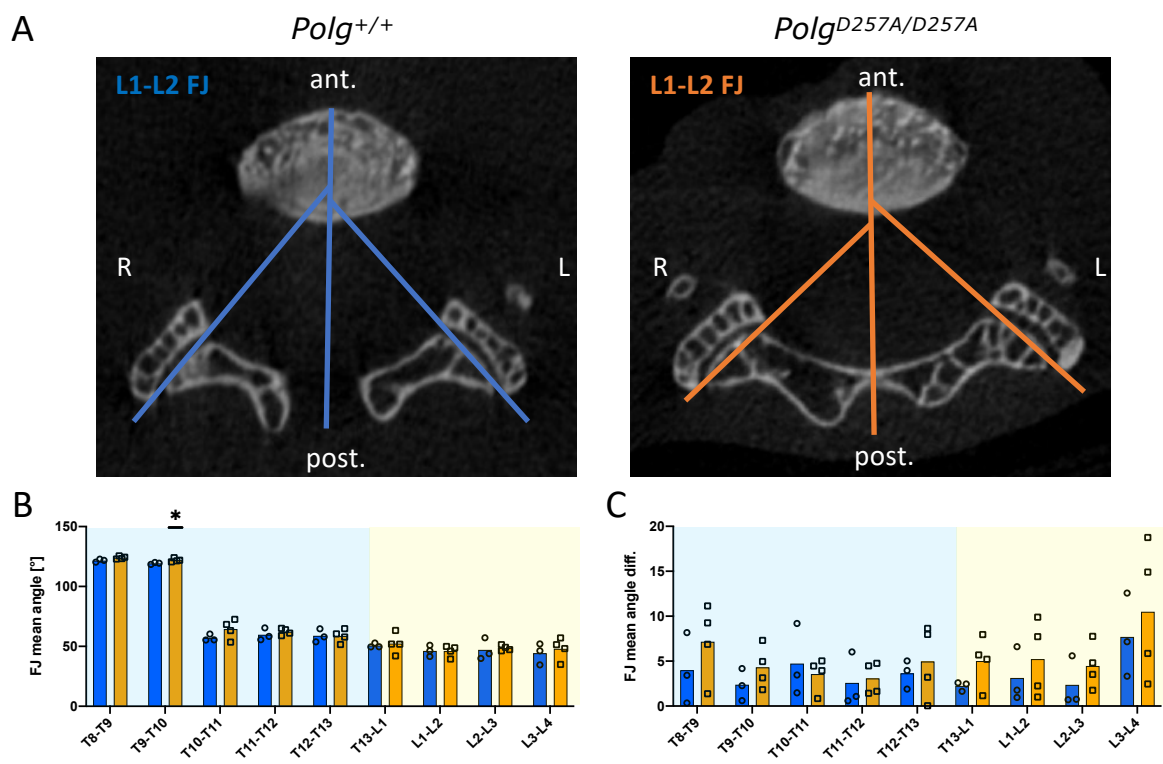

Supplement: Supplementary Fig. 4 — Comparative analysis of the transaxial orientation of the FJ at the T8-L4 spinal levels of wild type and mutant mice: two-dimensional transaxial μCT images of L1-L2 FJ with angle measurement according to Noren et al. (1991) (A); mean FJ angle (B); mean difference between right and left FJ angles (C). Data are expressed as fold over wild type (blue line), *p < 0.05 by Student's t-tests. [file mmc4.pdf]

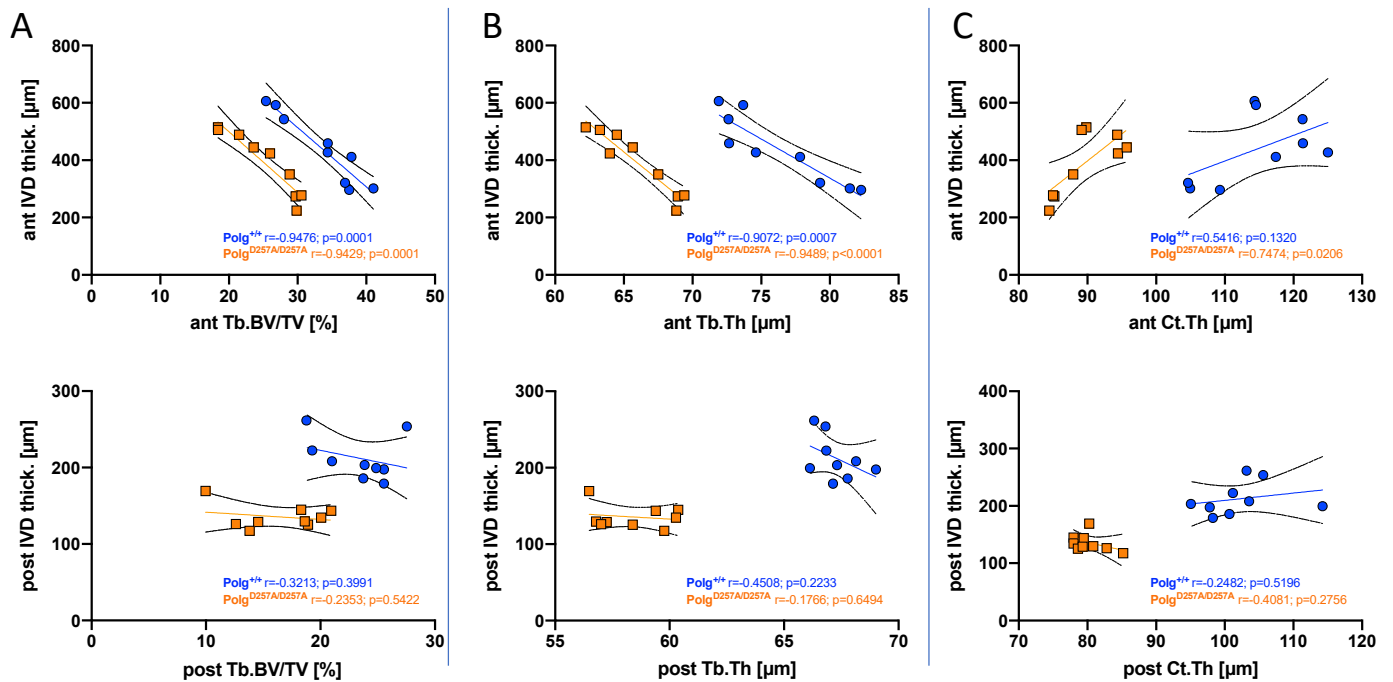

Supplement: Supplementary Fig. 5 — Pearson correlation analysis of mean values of bone parameters in the anterior (upper row) and posterior (lower row) portions of vertebrae (T8-L4) and their subjacent IVD anterior and posterior thickness, in wild type (blue circles) and homozygous (orange squares) mice: Tb.BV/TV and IVD thickness (A); Tb.Th and IVD thickness (B); Ct.Th and IVD thickness (C). Regression lines with 95 % confidence intervals, as well as correlation indexes and respective p values are displayed for wild types and homozygotes on each graph with corresponding colors. [file mmc5.pdf]
