# Supplementary material for: Polg mtDNA mutator mice reveal limited involvement of vertebral bone loss in premature aging-related thoracolumbar hyperkyphosis
Source: Bone Rep. 2022 Aug 30;17:101618. doi: 10.1016/j.bonr.2022.101618 (PMC9479024; doi:10.1016/j.bonr.2022.101618)
Supplement: Supplementary Fig. 3 — Comparative evaluation of average, anterior and posterior IVD thickness between wild type and mutant mice: Supplementary table with descriptive results from T8-L4 comparison with respective p values by Student's t-tests. [file mmc3.pdf]

Supp Fig 3

		average (T8-L4)			
		Mean	***SD***	***P value***	***t***
IVD. thick [µm]	*WT*	234.400	38.870	0.047	2.152
	Polg D257A	198.000	32.700		

		anterior (T8-L4)				***posterior (T8-L4)***			
		Mean	***SD***	***P value***	***t***	***Mean***	***SD***	***P value***	***t***
IVD. thick [µm]	*WT*	439.700	120.800	0.367	0.927	212.400	28.570	<0.0001	7.107
	Polg D257A	389.100	110.700			135.500	15.390		
